# *Fasciola hepatica*-Derived Molecules as Regulators of the Host Immune Response

**DOI:** 10.3389/fimmu.2020.02182

**Published:** 2020-09-02

**Authors:** Sinéad Ryan, Jenna Shiels, Clifford C. Taggart, John P. Dalton, Sinéad Weldon

**Affiliations:** ^1^Airway Innate Immunity Research (AiiR) Group, Wellcome-Wolfson Institute for Experimental Medicine, Queen’s University Belfast, Belfast, United Kingdom; ^2^Centre of One Health (COH), Ryan Institute, School of Natural Sciences, National University of Ireland Galway, Galway, United Kingdom

**Keywords:** parasite, helminth, helminth defense molecule, *Fasciola*, FhHDM-1, immunomodulation

## Abstract

Helminths (worms) are one of the most successful organisms in nature given their ability to infect millions of humans and animals worldwide. Their success can be attributed to their ability to modulate the host immune response for their own benefit by releasing excretory-secretory (ES) products. Accordingly, ES products have been lauded as a potential source of immunomodulators/biotherapeutics for an array of inflammatory diseases. However, there is a significant lack of knowledge regarding the specific interactions between these products and cells of the immune response. Many different compounds have been identified within the helminth “secretome,” including antioxidants, proteases, mucin-like peptides, as well as helminth defense molecules (HDMs), each with unique influences on the host inflammatory response. HDMs are a conserved group of proteins initially discovered in the secretome of the liver fluke, *Fasciola hepatica*. HDMs interact with cell membranes without cytotoxic effects and do not exert antimicrobial activity, suggesting that these peptides evolved specifically for immunomodulatory purposes. A peptide generated from the HDM sequence, termed FhHDM-1, has shown extensive anti-inflammatory abilities in clinically relevant models of diseases such as diabetes, multiple sclerosis, asthma, and acute lung injury, offering hope for the development of a new class of therapeutics. In this review, the current knowledge of host immunomodulation by a range of *F. hepatica* ES products, particularly FhHDM-1, will be discussed. Immune regulators, including HDMs, have been identified from other helminths and will also be outlined to broaden our understanding of the variety of effects these potent molecules exert on immune cells.

## Introduction

Helminths are parasitic worms classified as flukes, tapeworms or roundworms according to their appearance and the organ in which they reside during infection (1). Diseases caused by helminths constitute the majority of Neglected Tropical Diseases (NTDs) as classified by the World Health Organization (WHO). Helminths are one of the most successful infectious agents in nature as infection is highly prevalent and, as a result, over one billion people are affected worldwide (2, 3). One of the most prevalent zoonotic helminth diseases is fascioliasis caused by *Fasciola hepatica* and the larger *Fasciola gigantica*. This is a major foodborne disease that is currently thought to impact approximately two million people in over 70 countries, with developing countries more severely affected (4, 5).

The clinical manifestations of helminth infections are diverse; some infections elicit acute symptoms aligned with pathology caused by worm migration through host tissues, while others may be asymptomatic (6, 7). Co-evolution of humans and helminths may have shaped the human immune system as helminths developed sophisticated mechanisms to induce tolerance and evade expulsion by the host enabling them to become successful chronic pathogens (7–9). A range of genomic, transcriptomic, immunomic, glycomic, and proteomic approaches alongside database mining has provided further perspective on host-parasite interactions and led to the identification of various helminth molecules including those within excretory-secretory (ES) products that influence the host inflammatory response (10–15). These molecules have garnered much attention with the ultimate aim of exploiting their immunoregulatory mechanisms for the treatment of human diseases (16, 17). A number of molecules from *F. hepatica* and other worms are currently under investigation for immunotherapeutic potential and are the main focus of this review.

## Fasciola Hepatica

*F. hepatica* infection of humans and livestock occurs primarily through the consumption of encysted metacercariae. After ingestion, the metacercariae excyst and become newly excysted juveniles (NEJs) within the duodenum. What follows is the highly pathogenic and infectious migratory stage of *F. hepatica* infection where NEJs cross the intestinal wall to the liver via the peritoneum (18, 19). This phase is characterized by inflammation and damage until the NEJs reach the liver bile ducts where they mature into egg-producing adults. Different T cell responses and cytokine profiles observed in cells from the mesenteric (more IL-5) and hepatic lymph (more IL-4) nodes of mice infected with *F. hepatica* suggest that NEJ and hepatic-stage parasites produce different antigens that alter host responses (20). Despite initial inflammation, up to 50% of infected humans are asymptomatic (21). This is an extraordinary feat for any infectious agent as it indicates the ability to subvert the host immune response which is typically armed to expulse a pathogen. An increased abundance of IgG4 antibodies reactive to antigens (e.g., cathepsin L1) suggests a Th2-driven response is mounted (22); however, much of our knowledge of the immunology of fascioliasis is derived from ruminant animal infection and experimental models using rodents.

### Immunology of Fascioliasis

Helminth infestations often exist as chronic infections as a consequence of a Th2/regulatory response in the host that can support the survival and integrity of host tissue and the parasite (23, 24). The immune response mounted during the early stages of fascioliasis is generally regarded as a mixed Th1/Th2 response where cytokines such as IFNγ, IL-4, IL-10, and TGF-β are elevated. As the infection progresses, a Th2 response is amplified in conjunction with suppression of Th1 inflammation, thus allowing a prolonged infection that may be dependent on IL-4 (20). In the early stages of bovine *F. hepatica* infection, both IFNγ and IL-10 are increased, corroborating the idea that the initial immune response is mixed (25). Stimulation of peripheral blood mononuclear cells from cattle and sheep with *F. hepatica* ES products showed similar profiles (26, 27). In addition, TGF-β and IL-10 may modulate IL-4 and IFNγ in acute and chronic infection, respectively (28).

A cellular source of IL-10 was revealed in murine *F. hepatica* infection where, among increased macrophages and dendritic cells (DCs) in the peritoneal cavity, there was a significant population of CD25^+^Foxp3^+^ Treg and inducible Treg cells with the propensity to secrete IL-10 (29). Infection of IL-10^–/–^ mice showed that IL-4 and IFNγ responses were hindered by IL-10 (29). As IL-4 is a critical cytokine observed throughout the pathogenesis of *F. hepatica* infection, the appearance of an abundant population of alternatively activated macrophage (AAM) as early as 7 days after infection of mice in unsurprising (30). AAMs remain in the peritoneum for up to 3 weeks after oral infection with *F. hepatica* metacercariae, highlighting their key role in helminth disease (29). Eosinophilia in the peritoneum is evident in murine liver fluke infection (29) and bovine *F. hepatica* disease (31), and eosinophils contribute to tissue pathology, particularly in the liver (32). However, in sheep, eosinophils undergo apoptosis suggesting a mechanism by which *F. hepatica* evades the host response (33).

## *Fasciola Hepatica* Excretory-Secretory Products

**FIGURE 1:**
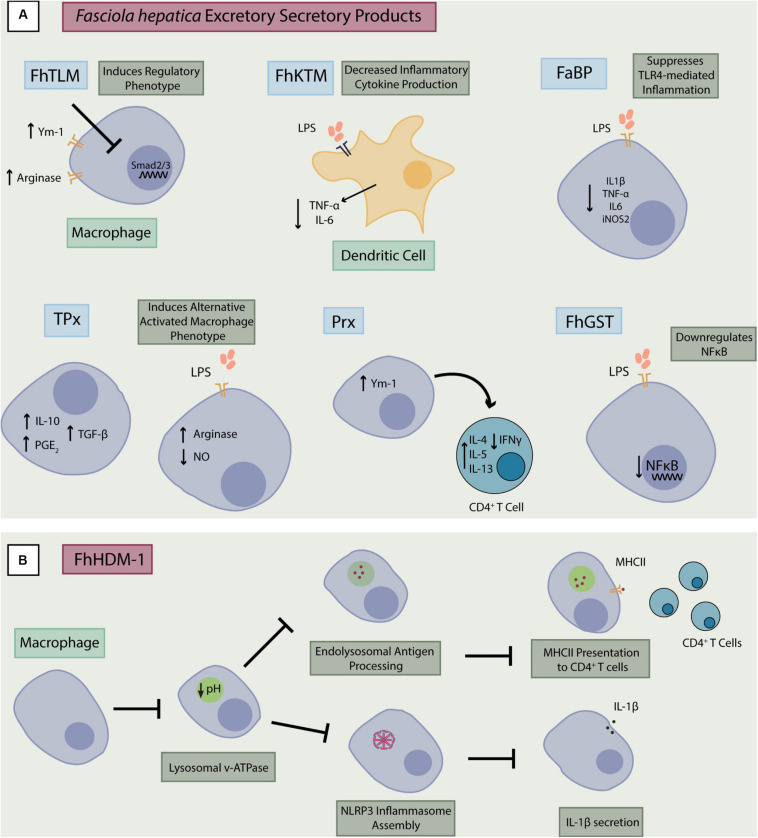
Immunomodulation by *F. hepatica* excretory-secretory products. **(A)** Excretory-secretory products from *F. hepatica* can modulate inflammatory responses in macrophages and dendritic cells in several ways. They may inhibit activity of NF-κB and the subsequent release of pro-inflammatory cytokines (TNF-α, IL-6, and IL-1β). They may also induce a regulatory phenotype through increased expression of factors such as Ym-1 or Smad2/3 which results in production of regulatory factors (IL-10 and TGF-β), augmentation of Th2 responses and suppression of Th1 inflammation. **(B)** FhHDM-1 interacts with macrophages and prevents lysosomal acidification which is necessary for antigen processing and major histocompatibility complex II (MHCII) presentation to T cells (68, 69). This action on the lysosome by FhHDM-1 also prevents assembly of the NLRP3 inflammasome, inhibiting release of IL-1β from the macrophage. FhTLM, *F. hepatica* TGF-like molecule; FhKTM, *F. hepatica* Kunitz-type molecule; FaBP, fatty acid binding protein; TPx, Thioredoxin peroxidase; Px, Peroxiredoxin, FhGST, *F. hepatica* Glutathione S-transferase; FhHDM-1, *F. hepatica* helminth defense molecule-1.

As parasites release ES products during host infiltration, it was deduced that they function as effector molecules capable of modulating the host immune system, enabling parasite survival. Various immunomodulatory molecules have been identified in the ES products of *F. hepatica* (Table 1) (34). Many of these molecules are advantageous to the helminth and, through manipulation of host immune processes, they facilitate prolonged parasitic infection. Anti-inflammatory effects (Figure 1) have been reported in rodent models of infection and inflammatory disease suggesting the potential for ES product development as therapeutics. However, many of the products discussed below are unique to certain life stages of the liver fluke leaving it difficult to define mechanisms without analysis of their purified or recombinantly produced forms.

**TABLE 1:** *F. hepatica*-derived immunomodulatory molecules.

Molecule	Abbreviation	Actions	References
Fatty acid binding protein	FaBP, Fh12, Fh15	Reduction of pro-inflammatory cytokines in LPS-induced models of sepsis	(44–46)
Helminth defense molecule	FhHDM-1	Inhibits lysosomal acidification and prevents macrophage antigen presentation Inhibits formation of the NLRP3 inflammasome and thus release of IL-1β Reduces inflammation in models of multiple sclerosis, type 1 diabetes, and allergic asthma	(53, 67, 69, 96)
Mucin	Fhmuc	Increases CD11b^+^MHCII^+^ macrophage during LPS stimulation and TLR4 expression is increased in DCs alluding to an increased Th1-type inflammatory response	(58, 59)
TGF-like molecule	FhTLM	Inhibits SMAD2/3 signaling and induces a regulatory phenotype in bovine macrophages	(64)
Kunitz-type molecule	FhKTM	Decreased inflammatory cytokine secretions in DCs	(56)
Glutathione S-transferases	FhGSTs	Suppress NF-κB pathway stimulation in macrophages and mice with endotoxemic shock have improved survival in the presence of GST treatment	(41, 42)
Thioredoxin Peroxidase/Peroxiredoxin	TPx/Prx	Induces Ym-1 expression and arginase activity in murine macrophages Antagonizes actions of ROS and induces AAM phenotype	(30, 35, 37)

### Antioxidants

The antioxidant enzymes thioredoxin peroxidase/peroxiredoxin (TPx/Prx) in *F. hepatica* ES products detoxify reactive metabolites produced by the host (35, 36). *F. hepatica* ES products induced AAMs and TPx did so without traditional Th2 signaling, i.e., IL-4 or IL-13 (30, 37). Administration of purified TPx to BALB/c mice induced a Th2 response as well as expression of Ym-1, TGF-β, and IL-10, and release of prostaglandin E_2_ (PGE_2_) from murine macrophages (30, 37). Glutathione S-transferases (GSTs) constitute up to 4% of the total protein in *F. hepatica* ES products and protect the helminth from free radicals that arise from the host response mounted to expulse the worm (38–40). In DCs, recombinant Sigma-class GST (rFhGST-si) interacts with TLR4 to stimulate IL-6 and MIP-2 production and CD40 expression via mitogen-activated protein kinase (MAPK) and NF-κB activity (41). Crucially, rFhGST-si inhibited development of Th17 cells without any interaction with the Th2-type response (41). Although recombinant Mu-class GST isoforms (rFhGST-mu) had no effect on DC activation (41), anti-inflammatory properties of native FhGST-mu (nFhGST-mu) were recently identified in monocytic cells stimulated with a range of TLR agonists and bacteria such as *Klebsiella pneumonia*, and treatment protected mice from endotoxemia (41, 42). In addition, nFhGST-mu suppressed the NF-κB pathway possibly via JAK/STAT signaling proteins and thus it was proposed that nFhGST may be a key antigen utilized by *F. hepatica* to suppress Th1 responses (42).

### Fatty Acid Binding Proteins

*F. hepatica* fatty acid binding proteins (FaBPs) are a group of chaperones that mediate lipid responses within the cell and are closely linked with inflammation and metabolism (43). Four FaBPs identified in *F. hepatica* are known antioxidants with a nutritive role for the parasite (44). Investigations into their anti-inflammatory properties demonstrated that the 12 kDa Fh12 product reduced pro-inflammatory cytokine production in the LPS-induced model of murine sepsis (45). Similarly, the 14.5 kDa Fh15 molecule attenuated production of IL-1β and TNF-α in human THP-1 macrophages (46). In a murine model of sepsis, Fh15 treatment was associated with a significant decrease in circulating cytokines (46).

### Cysteine Proteases

Cysteine proteases constitute approximately 80% of the ES products from *F. hepatica* and they play major roles throughout infection (47). Five clades of *F. hepatica* cathepsin L (FhCL) have been identified; three associated with mature adult worms (FhCL1, FhCL2, and FhCL5) and two specific to infective juvenile stage (FhCL3 and FhCL4). Increased secretion of FhCL3 during the initial stages of infection aid the immature NEJ by preventing attachment of host eosinophils (48). Conversely, once the fluke has reached the liver, FhCL1/2 secretions elicit anti-coagulant effects that allow blood feeding for the parasite (49). FhCL1 dampened the Th1 response elicited by administration of the *Bordetella pertussis* vaccine in mice (50). The decrease in the IFNγ response concurs with previous evidence that concurrent *F. hepatica* and *B. pertussis* infection had a decreased Th1-centric response (51). Interestingly, the effects of FhCL1 translated into decreased inflammatory mediators and protective effects in LPS-induced septic shock (52). Although recombinant FhCL1 partially activated DCs via TLR4, these DCs suppressed the development of Th17 cells and did not induce the differentiation of Th2 cells (41). Hypo-responsiveness in peritoneal macrophages stimulated with LPS and FhCL1 indicated that MyD88-independent/TRIF-dependent signaling through cleavage of TLR3 in the endosome was inhibited (52). However, in murine models of type 1 diabetes (T1D) and multiple sclerosis, FhCL1 treatment showed no benefit (53).

### Protease Inhibitors

Kunitz serine protease inhibitors have been identified in the total extract and tegument of *F. hepatica* (54). Interestingly, *F. hepatica* Kunitz type molecule (FhKTM) has an unique specificity for cysteine proteases (13) and was shown to associate with cathepsin L (55). FhKTM induced a regulatory IL-27-dependent phenotype in LPS-stimulated DCs that impaired Th1 and Th17 responses (56).

### Mucin-Like Peptides

Analysis of the NEJ stage of *F. hepatica* infection led to the discovery of proteins with similarities to mucins (57, 58). A synthetic mucin-derived peptide (Fhmuc) increased peritoneal CD11b^+^MHCII^+^ cells in mice exposed to LPS (59). In contrast to other *F. hepatica* ES products discussed here, but similar to FhCL1 and FhGST-si (41), Fhmuc elicits pro-inflammatory properties, with increased LPS-induced TLR4 expression in DCs and polarization of the T cell response (59). This ability of *F. hepatica* to modulate the host immune response may have potential implications for vaccination strategies (59). As *F. hepatica* ES products contain significant levels of glycans, it is probable that native *F. hepatica* mucin-like peptides undergo glycosylation, which is not represented with the synthetic peptide. Thus, the immunomodulatory effects of native Fhmuc might be different to those described for the synthetic peptide. Indeed, Rodríguez et al. have shown that glycans from *F. hepatica* modulate DC function to induce a Th2 response and suppress Th1 inflammation (60–62).

### TGF-β Mimics

Three distinct TGF-β homologs were identified in *F. hepatica* through bioinformatic approaches (63). *F. hepatica* activin/TGF-like molecule (FhTLM) is highly conserved with other TGF-β homologs from nematode parasites and has a limited temporal expression pattern across parasite development (63). Recombinant FhTLM supported NEJ viability and development (63). FhTLM may be less potent than mammalian TGF-β, however, SMAD-2/3 signaling characteristic of the regulatory phenotype was observed in bovine macrophages as well as alternative activation (64).

### Helminth Defense Molecules (HDMs)

During helminth infiltration, increased bacterial infection is common as a consequence of the characteristic tissue damage, although the host inflammatory response remains stable. In schistosomiasis, enteric bacteria are displaced during destruction of the gut; however, symptoms of infection or sepsis are not apparent suggesting the host may be immunosuppressed by the parasite (65). Thus, it was hypothesized that parasites secrete antimicrobial peptides (AMPs) similar to those released by the host as a protective mechanism during infection. Investigation of *F. hepatica* ES products discovered an 8 kDa protein constitutively expressed at all stages of the life-cycle and throughout infection (14). BLAST analyses indicated the sequence was conserved throughout trematode helminth species including *Paragonimus westermani*, *Schistosoma mansoni*, and *Schistosoma japonicum*, and thus was named the helminth defense molecule (HDM). HDMs are classified into three clades: Schistosome HDMs, Fasciola/Asian fluke HDMs and Sm16-like molecules. All clade members have a predicted N-terminal peptide and α-helical structure as well as a highly conserved, largely hydrophobic C-terminal sequence of approximately 35 residues (14).

## *F. Hepatica* Helminth Defense Molecule-1 (FhHDM-1)

The first discovered HDM was from *F. hepatica* (FhHDM-1, Figure 1) (14). FhHDM-1 is predicted to have a predominantly α-helical secondary structure with a C-terminal amphipathic helix bearing structural and biochemical resemblance to mammalian cathelicidins. Moreover, the 34 residue C-terminal sequence from FhHDM-1 has striking similarity to the human cathelicidin, LL-37 (14, 66, 67). Like LL-37, FhHDM-1 and its conserved C-terminal fragment neutralize LPS preventing TLR4 activation on target cells such as macrophages (14, 68). However, in a murine model of intratracheal LPS-induced acute lung injury, intraperitoneal administration of FhHDM-1 decreased neutrophilic lung inflammation, suggesting mechanisms beyond LPS neutralization may be involved (69). Perhaps unexpectedly, FhHDM and other HDMs did not elicit any antimicrobial activity against different bacteria such as *Escherichia coli*, *Pseudomonas aeruginosa*, and *Staphylococcus aureus* (70). However, in contrast to the mammalian AMPs, they did not induce pore formation in macrophages or the release of lactate dehydrogenase indicating that HDMs do not elicit cytotoxic effects.

The immunomodulatory role of FhHDM-1 was investigated in models of inflammatory disease where ES products showed promise as anti-inflammatories. *F. hepatica* ES products reduced inflammation in the non-obese diabetic (NOD) T1D mouse model that correlated with an increase in M2 macrophages and Foxp3^+^ Tregs (71). Administration of a synthetic FhHDM-1, but not FhCL1, in NOD mice had a comparable effect with a decreased disease burden characterized by improved survivor function and fewer mice developing diabetes (53). While destruction of pancreatic β cells is mediated mainly by autoreactive T cells (72), inhibition of macrophage activity by FhHDM-1 may elicit positive effects on clinical measurements (53). *F. hepatica* total extract, ES products and FhHDM-1 have proven beneficial in other autoimmune diseases, for example in the murine experimental autoimmune encephalomyelitis (EAE) model of multiple sclerosis (53, 73, 74). A predominant theory behind the activity of helminth ES products is their ability to induce a regulatory Th2 response that is often characterized by a M2 (AAM) phenotype in macrophages. While FhHDM-1 reduced TNF-α and IL-6 secretion in LPS-stimulated macrophages (53), there were no significant alterations in surface receptor expression or release of Th1 suppressing cytokines, such as TGF-β and IL-10. Furthermore, the auto-antigen specific T cell response remained unchanged in EAE mice that received FhHDM-1 despite showing reduced disease severity (53). This implies that mechanisms other than an induced Th2 response may be responsible for decreased Th1/Th17-mediated pro-inflammatory activity.

FhHDM-1 associates with lipid rafts in the macrophage plasma membrane and is endocytosed (67). Within the macrophage, FhHDM-1 is cleaved by lysosomal cathepsin L to release a C-terminal peptide that can form an amphipathic helix, and this peptide prevented acidification of the lysosomes through inhibition of vacuolar ATPase (vATPase) activity (67). Macrophage process antigens and present them to MHC-II on CD4^+^ T cells; therefore, impairment of this process via vATPase inhibition would prevent initiation of the adaptive immune response (67). To further define FhHDM-1 mechanisms, Donnelly and colleagues hypothesized that inhibition of lysosomal activity in the macrophage (75) would impact on inflammasome activity and release of pro-inflammatory IL-1β (76). Indeed, it was observed that FhHDM-1 reduced IL-1β release in macrophages stimulated with the NLRP3 activator NanoSiO_2_ and alum. The cysteine protease cathepsin B is a pH-dependent lysosomal protease involved in activation of the NLRP3 inflammasome (76). As FhHDM-1 inhibits lysosomal acidification, it would inhibit cathepsin B activity and the NLRP3 inflammasome. Replicates of these experiments carried out using the 34 residue C-terminal sequence of FhHDM-1 indicate that these effects on the inflammasome are unique to FhHDM-1 (76). Gene expression analysis of macrophages stimulated with LPS predicted that signaling associated with high-mobility group box-1 (HMGB-1) and IL-17 were attenuated by FhHDM-1 (69). This posed the question of whether FhHDM-1 nay be effective in preventing allergic inflammation, which was subsequently tested in a rodent model of house dust mite-induced asthma (69). In this model, FhHDM-1 treatment reduced neutrophil and eosinophil cell counts, inflammatory markers and airway mucus content (69).

## What Have We Learned From Other Helminth Excretory-Secretory Products?

Immunomodulatory functions and modes of action have been outlined for ES products from several parasites. The filarial worm *Acanthocheilonema viteae* releases a glycoprotein called ES-62 that can interfere with DC TLR4 expression by inducing autophagosomal degradation (77). By inhibiting mast cell responses, ES-62 and its small molecule analogs prevented the excessive inflammatory response in murine models of asthma (78, 79). However, ES-62 also controls the Th1 response by suppressing NF-κB-mediated inflammation in DCs (80). More recently, ES-62 was found to inhibit IL-33/ST2/MyD88 signaling and modulate the pro-inflammatory responses resulting from crosstalk between ST2, FcεRI, and TLR4, which may contribute to reported protective effects of ES-62 in chronic models of asthma (81).

Mice with a gastrointestinal infiltration of *Heligmosomoides polygyrus* have characteristic increases in the number of Treg (CD4^+^CD25^+^) cells. ES products from this parasite potentiate the expression of Foxp3 in CD4^+^ T cells *in vitro* through mimicry of TGF-β (82). These findings led to the discovery of *H. polygyrus* TGF-β mimic (Hp-TGM) that operates through traditional TGF-β signaling pathways leading to polarization of CD4^+^ T cells with potent suppressive abilities (83). The recently identified *H. polygyrus* alarmin release inhibitor (HpARI) binds to active IL-33 preventing its interaction with ST2 both in murine and human models, and could provide novel therapeutic options in Th2 dominated disease, such as asthma (84).

*S. mansoni* ES products have pleiotropic effects on the immune response. Factors released from the parasite and its eggs can modulate both Th1 and Th2 responses. For example, IPSE (IL-4-producing principle from schistosome eggs) expanded the population of regulatory B cells, which in turn activated Tregs via IL-10 (85) and *Schistosoma haematobium* IPSE showed therapeutic efficacy in a murine model of hemorrhage in the bladder (86). *S. mansoni* chemokine binding protein (SmCKBP) is secreted from live eggs and can bind and neutralize the neutrophil chemoattractant CXCL-8 (87). In an experimental granulomatous inflammation model, blocking of live egg smCKBP increased recruitment of neutrophils, macrophage and eosinophils and the size of the egg granuloma, suggesting this ES product may limit leukocyte recruitment to protect the egg (87).

## Concluding Remarks

The success of *F. hepatica* infection stems from the worm’s ability to modify and manipulate the host immune response. While many years of research have uncovered effective mechanisms by which the parasite can establish a long-term infection, there is much more to be revealed. Investigations in various models of inflammatory disease indicated potential therapeutic benefit of helminths and ES products, however, to-date the majority of human clinical trials have not replicated these findings (11, 88–91). While small animal models of inflammatory disease provide valuable insights, they often fail to recapitulate the various complex processes at play in human conditions (91). There are a number of factors that need to be considered such as differences in metabolism and the impact of microbiota, particularly on the immune response (92), that may affect efficacy of helminths and their products. Nonetheless, clinical trial outcomes have highlighted the need for a greater understanding of the complexity of changes to the immune response induced by helminths during infection.

A typical helminth genome contains around 50,000 genes, which is much greater than the human genome (approximately 20,000), and it has been proposed that each parasite has undergone specific adaptations for their particular niche (93). Furthermore, as helminths have a multistage life cycle with distinct developmental stages through select tissues and organ systems, they may release distinct molecules within a particular niche (e.g., the intestine or lung) or migratory stages that exert more localized immunomodulatory effects (94), which may be of relevance for targeting tissue-specific inflammation. As recently reviewed by Cortés et al. (95) and van der Zande et al. (9), a number of studies have identified potential roles for helminth-host microbiome interaction in the pathophysiology of helminth disease and in parasite-mediated suppression of host inflammation, which may be relevant for the targeting of gut and lung inflammation and (immuno)metabolic dysfunction. In addition to the protein molecules outlined herein, there are a number of other families of helminth immunomodulators, which include various carbohydrate, nucleotide and lipid mediators as well as extracellular vesicles that require further investigation (16). Better understanding of the individual components of helminth ES products and in-depth characterization of their functional roles using defined products may help shed further light on their potential efficacy as therapeutic or prophylactic agents for human disease. The discovery and characterization of HDMs from *F. hepatica* and other trematodes may provide one such avenue for novel therapeutics for autoimmune and inflammatory conditions. While further work is needed to better define these molecules, their host targets and their functional effects, there is an expectation that this work will spark the development of novel biotherapeutics for an array of inflammatory diseases.

## Author Contributions

All authors listed have made a substantial, direct and intellectual contribution to the work, and approved it for publication.

## Conflict of Interest

The authors declare that the research was conducted in the absence of any commercial or financial relationships that could be construed as a potential conflict of interest.
